# CD8^+^ T Cells and IFN-γ Mediate the Time-Dependent Accumulation of Infected Red Blood Cells in Deep Organs during Experimental Cerebral Malaria

**DOI:** 10.1371/journal.pone.0018720

**Published:** 2011-04-11

**Authors:** Carla Claser, Benoît Malleret, Sin Yee Gun, Alicia Yoke Wei Wong, Zi Wei Chang, Pearline Teo, Peter Chi Ee See, Shanshan Wu Howland, Florent Ginhoux, Laurent Rénia

**Affiliations:** Singapore Immunology Network (SIgN), Agency for Science, Technology and Research (A*STAR), Biopolis, Singapore, Singapore; State University of Campinas, Brazil

## Abstract

**Background:**

Infection with *Plasmodium berghei* ANKA (PbA) in susceptible mice induces a syndrome called experimental cerebral malaria (ECM) with severe pathologies occurring in various mouse organs. Immune mediators such as T cells or cytokines have been implicated in the pathogenesis of ECM. Red blood cells infected with PbA parasites have been shown to accumulate in the brain and other tissues during infection. This accumulation is thought to be involved in PbA–induced pathologies, which mechanisms are poorly understood.

**Methods and Findings:**

Using transgenic PbA parasites expressing the luciferase protein, we have assessed by real-time *in vivo* imaging the dynamic and temporal contribution of different immune factors in infected red blood cell (IRBC) accumulation and distribution in different organs during PbA infection. Using deficient mice or depleting antibodies, we observed that CD8^+^ T cells and IFN-γ drive the rapid increase in total parasite biomass and accumulation of IRBC in the brain and in different organs 6–12 days post-infection, at a time when mice develop ECM. Other cells types like CD4^+^ T cells, monocytes or neutrophils or cytokines such as IL-12 and TNF-α did not influence the early increase of total parasite biomass and IRBC accumulation in different organs.

**Conclusions:**

CD8^+^ T cells and IFN-γ are the major immune mediators controlling the time-dependent accumulation of *P. berghei*-infected red blood cells in tissues.

## Introduction

Malaria remains one of the most health-threatening diseases worldwide and is a major global cause of morbidity and mortality, with ∼800 000 deaths worldwide each year [1], [2]. Patients with severe *Plasmodium falciparum* malaria develop complications like acidosis, edema, respiratory problems, jaundice, hypoglycemia and cerebral malaria (CM) [3]. CM is the most severe complication of *P. falciparum* infection, afflicting primarily children aged 2–6 years in sub-Saharan Africa, adults in Southeast Asia and pregnant women [3]. Clinically, it is defined as a diffuse encephalopathy causing unrousable coma often associated with seizures in the presence of asexual forms of *P. falciparum* in peripheral blood and not attributable to other causes of unconsciousness [3]. The mechanisms leading to CM in humans are not completely understood and two main hypotheses, one mechanical and one immunological, have been proposed and are still actively debated [4]–[6]. The mechanical hypothesis is based on the observation that mature infected red blood cells (IRBC) bind to the endothelium of the brain and other organs [7]. IRBC cytoadherence is responsible for the intravascular sequestration of mature asexual forms in tissues and their disappearance from the peripheral circulation [8]–[10]. Thus, it has been proposed that accumulation of sequestered IRBC causes obstruction of brain microvessels leading to decreased blood flow, hypoxia, hemorrhages, coma and death [10]. However, it has been reported that some patients clinically diagnosed with human cerebral malaria had little or no IRBC sequestered in their brain capillaries [11], [12], suggesting that IRBC sequestration might not be sufficient per se to cause CM. The immunological hypothesis is based on the findings that i) a strong inflammatory response, characterized by elevated levels of pro-inflammatory cytokines [13], [14], is observed during the acute phase of *P. falciparum* infection and ii) leukocytes and platelets, together with IRBC, were found sequestered intravascularly not only in the brain microvessels of CM patients [15], [16], but also in other organs such as the lungs [11]. IRBC and malaria toxins stimulate immune cells to produce large amounts of pro-inflammatory cytokines such as TNF-α, IFN-γ and lymphotoxin-α [6]. These inflammatory cytokines induce the production of chemokines and up-regulate the expression of some adhesion molecules, such as ICAM-1 and/or VCAM-1 on endothelial cells in ECM [17]. Chemokines are responsible for the attraction of leukocytes and platelets to the brain and other tissues, where adhesion molecules mediate the cytoadherence and sequestration of these immune cells in the brain and other organs. Inflammation-induced adhesion molecules of the endothelium, such as ICAM-1, can also act as ligands for cytoadherent IRBC [18], [19], thus bridging the mechanical and the immunological hypotheses.

Ethical concerns limit the analysis of pathogenic mechanisms leading to CM at the tissue level. Therefore many mechanistic studies have relied on the use of rodent malaria models [20] which allow access to deep tissues. Susceptible mice infected with *P. berghei* ANKA develop a severe syndrome with cerebral pathology leading to coma and death. This experimental cerebral malaria (ECM) model is characterized by the intravascular accumulation of immune cells in the brains of mice which die during the first 10 days of infection with signs of neurological involvement [20] and evidence of circulatory shock [21]. Different cell subsets such as monocytes, NK, neutrophils and T cells have been shown to migrate to the brain coincident with the neurological signs of ECM [22], [23]. CD8^+^ T cells have been shown to be the primary effectors of ECM [22], whereas NK and CD4^+^ T cells have been implicated in the induction phase of ECM [22], [23]. Pro-inflammatory cytokines such as IFN-γ [24] and lymphotoxin-α [25] are also essential for ECM to occur.

The precise role of IRBC accumulation in ECM pathogenesis is still unknown. PCR-based methods were first used to demonstrate that parasites accumulate in the brain during infection [26], [27]; this was subsequently confirmed by histological studies [28]. Recently, dynamic investigation of IRBC distribution in organs during the course of infection has been made possible by bioluminescence imaging using transgenic PbA parasites expressing luciferase [29]. The pioneer study by Franke-Fayard *et al.* [30] detected IRBC accumulation in various organs during infection but concluded that ECM was unrelated to IRBC accumulation because of the low signal in the brain. However, recent studies [31], and our unpublished results have clearly demonstrated IRBC accumulation in the brain when imaging parameters were optimized. Whereas there is considerable evidence that IRBC sequestration induces pro-inflammatory cytokine and chemokine secretion and the recruitment of immune cells (reviewed in [6]), whether inflammation and immune cells in turn contribute to IRBC accumulation remains an unresolved question. In this paper, we report the results of our bioluminescence imaging experiments showing that CD8^+^ T cells and IFN-γ drive IRBC accumulation in the brain and also in other organs only for the duration of ECM.

## Materials and Methods

### Ethics

All experiments and procedures were approved by the Institutional Animal Care and Use Committee (IACUC) of A*STAR (Biopolis, Singapore) (Authorization No IACUC 080321) in accordance with the guidelines of the Agri-Food and Veterinary Authority (AVA) and the National Advisory Committee for Laboratory Animal Research (NACLAR) of Singapore.

### Mice

Female or male wild-type (WT) and CD8^−/−^, CD4^−/−^, Rag2^−/−^, CCR2^−/−^, IFN-γ^−/−^, IL12p40^−/−^, and TNFα^−/−^ deficient C57BL/6J mice were used. MAFIA (macrophage Fas-induced apoptosis) mice on a C57BL/6J background [33] were also used. All mice (7–8 wk old) were bred and kept under specific pathogen-free conditions in the Biomedical Resource Centre, Singapore.

### Parasite infection and evaluation of the disease

The transgenic *Plasmodium berghei* ANKA (231c1l) line expressing luciferase and green fluorescent proteins under control of the ef1-α (PbA*luc*) used in this study was kindly provided by Dr. Christian Engwerda (QIMR, Brisbane, Australia) [30], [31]. IRBC stabilates used to initiate infections were free from other infectious agents and were prepared through *in vivo* passage in C57BL/6J mice and stored in liquid nitrogen (10^7^ IRBC/ml in Alseveer's solution) [34]. All mice were infected intraperitoneally with 10^6^ IRBC. Parasitemia was monitored daily by flow cytometry starting from day 3 till day 12, and afterwards every 2 days till day 30. From day 3 to day 5, 5×10^5^ cells were acquired and from day 6 onwards, 10^5^ cells. Sixty- to 100% of susceptible mice of the C57BL/6J background developed ECM depending on the experiments and on the parasite batch used to initiate infection. Mice were considered to have ECM if they displayed the following neurological symptoms: paralysis, ataxia, deviation of the head and convulsion and/or coma. The mice that did not develop ECM died of hyperparasitemia and anemia later in the infection.

### *In vivo* and *ex vivo* imaging

IRBC accumulation in mice infected with PbA*luc* was assessed daily using an *in vivo* imaging system (IVIS, Xenogen, Alameda, Ca). The luciferase substrate, D-luciferin potassium salt (Caliper Life sciences), was dissolved in phosphate-buffered saline at a concentration of 5 mg/ml. Mice were anesthetized in an oxygen-rich induction chamber with 2% isoflurane and shaved, then measurements were performed 2 min after subcutaneous injection of 100 µl of luciferin. Whole body imaging was performed with the animal in the ventral position (facing the camera) (Figure S1A). Head imaging was performed on the animal in the dorsal position (with the back of their head facing the camera (Figure S1B). Bioluminescence imaging was acquired with 21.7 and 4 cm FOV for whole body and head respectively, medium binning factor, and exposure time of 5–60 s (changed according to the intensity of the bioluminescence signal). For some experiments, individual organs were removed from intracardially perfused or non-perfused mice, 3 min after a second subcutaneous injection of luciferin. Organs were placed in 24 well plates and imaged with 10 cm FOV, medium binning factor, and exposure times of 10–60 s. To allow comparisons between images from different days, uninfected mice injected with luciferin were imaged to for background subtraction. For bioluminescence quantification, regions of interest (ROI) were drawn by using the software Living Imaging 3.0 and average radiance (p/s/cm^2^/sr) was determined.

### *In vivo* leukocyte subpopulation and macrophage depletion

Purified rat IgG2b anti-mouse CD8 (clone 2.43; TIB 210; American Type Culture Collection (ATCC), Manassas, VA) and rat IgG2a anti-mouse CD4 (clone GK1.5; TIB 207; ATCC) mAbs were used from stocks previously shown to efficiently deplete the relevant T cell subsets [35]. A total of 1 mg of mAbs was injected intraperitoneally at day 6 after parasite infection, before the onset of CM. The non–depleting anti-mouse M-CSF receptor mAb (AFS98) [36] was used to prevent monocyte accumulation into tissues [37], [38] by injecting 2 mg intraperitoneally at day 5 and 6 post-infection. For monocyte/granulocyte depletion in MAFIA mice, the nontoxic, cell-permeable small dimerizer drug AP20187 (a gift from Ariad Pharmaceuticals) was used. A stock solution of 13.75 mg/ml AP20187 in ethanol was diluted to 0.55 mg/ml in 4% ethanol, 10% PEG-400, and 1.7% Tween in water. The mice were intraperitoneally injected at day 5, 6 and 7 after parasite infection, with a volume adjusted according to mouse body weight to deliver 10 mg AP20187 per kg mouse. It has to be noted that CD115 has been shown to be expressed in microglial cells [39]. However, although the inducible suicide transgene was clearly expressed in resident brain macrophages, treatment with the dimerizing agent did not induce deletion of microglial cells (identified here as CD45-CD11b+) in our experiment or in previous studies [33]. CD115 is also expressed by neurons in mouse brain [40]. However, treatment of normal MAFIA mice with the dimerizing agent did not induce any neurological manifestations or alterations [33]. This is probably due the limited capacity of the dimerizing drug to cross the blood-brain barrier and access brain parenchyma.

### Purification of tissue and blood leukocytes

Mice were perfused intracardially with PBS to remove circulating RBC, IRBC and leukocytes. Organs were removed and processed as previously described [22]. For the blood, 50 to 100 µl was collected into PBS 10 µM EDTA to prevent clotting. Red blood cells were removed by incubation in a buffered ammonium chloride (ACK) solution and cells washed with FACS buffer (PBS 1% BSA 2 µM EDTA). Pellet was resuspended into 100 µl blocking buffer (FACS buffer containing 1% rat and mouse serum). After 30 minutes incubation on ice, cells were stained for FACs analysis. For live/dead discrimination, cells were resuspended in 200 to 300 µl FACS buffer containing DAPI (0.1 µg/ml).

### Flow cytometry analysis

Flow cytometry studies were performed using LSR II (Becton Dickinson) and subsequently analyzed using the FlowJo software (Tree Star). Fluorochrome or biotin-conjugated monoclonal antibodies (mAbs) specific for mouse CD11b (clone M1/70), CD45 (clone 30F11), CSF-1R (or CD115) (clone AFS98), Gr-1/Ly6C/G (clone RB6-8C5), the corresponding isotype controls and the secondary reagents (phycoerythrin coupled to streptavidin) were purchased either from BD Biosciences or e-biosciences. Anti-F4/80 (A3-1) mAb was purchased from Serotec. Analysis was carried out by gating on singlets DAPI– (4,6-diamidino-2-phenylindole) CD45^+^ cells.

### Statistical analysis

Difference in survival was assessed with the log-rank Kaplan-Meier test. Differences between two groups were analyzed for statistical significance using the Mann-Whitney *U* test and for multiple groups using one-way ANOVA and the Dunn post-test. The data were analyzed using GraphPad Prism software (version 3.0) and taking *p*<0.05 as the level of significance.

## Results

### CD8^+^ T cells regulate parasite accumulation in the brain during ECM

Since immune mediators such as T cells and cytokines have been shown to be crucial for ECM development, we tested the hypothesis that parasite accumulation in the brain and other organs is modulated by the immune system.

**Figure 1:**
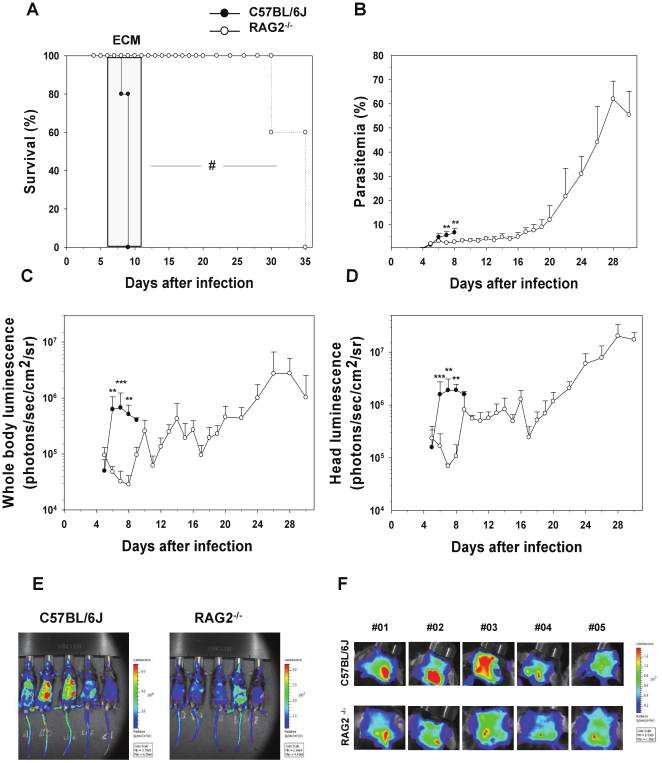
Decrease in parasite biomass and IRBC accumulation in organs in infected Rag2^−/−^ mice. A) Survival and (B) parasitemia in WT (*n* = 5) and Rag2^−/−^ mice (n = 5) infected with PbA*luc*. Neurologic signs of CM appeared on days 6–12 (shaded area), with death occurring 24–48 h after onset. Parasitemia (%) values are expressed as mean ± SD of 5 mice per group. *In vivo* bioluminescence imaging quantification of IRBC accumulation in the whole body (C) and head (D) of infected WT and Rag2^−/−^ mice. Mice were injected with luciferin and IRBC accumulation was measured by bioluminescence imaging as described in Materials and Methods. Each bar represents mean luminescence values (log) ± SD of 5 mice. #p<0.01, log-rank test and **p<0.01 and ***p<0.001, Mann Whitney test. Pseudocolor images of whole body (E) and Head (F) from infected WT and RAG2 KO mice.

**Figure 2:**
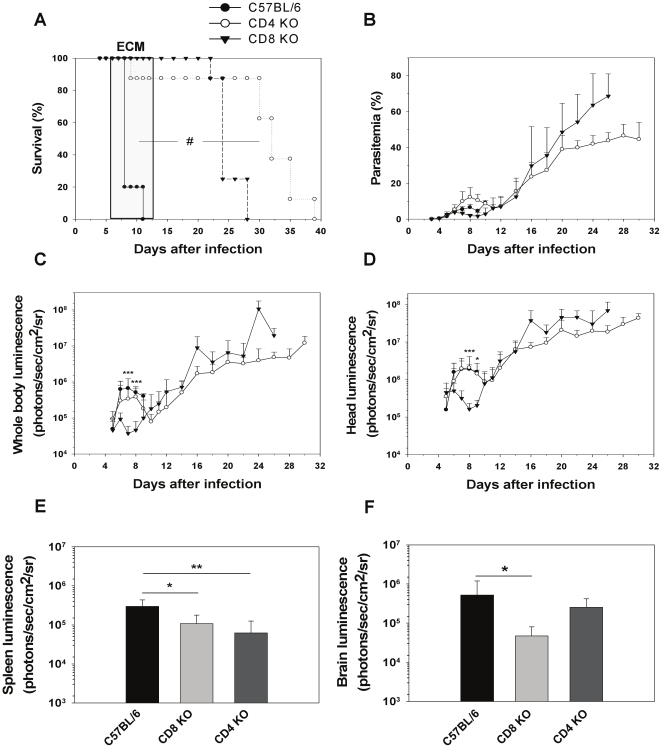
Decrease in parasite biomass and IRBC accumulation in organs in infected CD8^−/−^ mice. A) Survival and (B) parasitemia of CD8^−/−^ (*n* = 8), CD4^−/−^ (*n* = 8) and WT (*n* = 10) mice infected with PbA*luc*. Neurologic signs of CM appeared on days 6–12 (shaded area), with death occurring 24–48 h after onset. Parasitemia (%) values are expressed as mean ± SD. *In vivo* bioluminescence imaging quantification of IRBC accumulation in the whole body (C) and head (D) of infected mice. *Ex vivo* quantification by bioluminescence imaging of IRBC accumulation in spleens (E) and brains (F) obtained from five perfused mice per group. Luminescence values (log) as mean ± SD. #p<0.01, log-rank test; *p<0.05, **p<0.01 and ***p<0.001, ANOVA followed by Bonferroni test.

First, we assessed the role of the adaptive immune system using RAG-2^−/−^ mice which lack B and T cells. RAG2^−/−^ mice infected with PbA*luc* did not develop ECM contrary to wild-type (WT) C57BL/6J mice which died with neurological signs 6 to 12 days post-infection (Figure 1A). They had significantly lower parasitemias in peripheral blood than the WT mice during the first week of infection and survived past the third week of infection, eventually dying of anemia and hyperparasitemia (Figure 1B). Parasite biomass was monitored in parallel by bioluminescence imaging in WT and RAG2^−/−^ mice. Strikingly, a strong increase in parasite biomass was observed in WT mice when they developed patent ECM signs (Figure 1C and 1E). In contrast, total parasite biomass (determined by imaging the whole body) remained low in the RAG2^−/−^ mice until it increased steadily from day 9 onwards, paralleling the increase in parasitemia. A similar result was observed when parasite biomass measurement was restricted to the head (Figure 1D and 1F). This demonstrated that total and head parasite biomasses are under the control of T or B cells during the time frame of PbA development and is clearly associated with the development of ECM. Since previous studies using B-deficient mice showed that these cells were not involved in ECM pathogenesis [41], we turned our attention to mice lacking CD4 and CD8 T cells. Eighty five percent of CD4^−/−^ mice and all of the CD8^−/−^ mice failed to develop ECM (Figure 2A) even though parasitemias were similar to those of WT mice during the first week of infection (Figure 2B). Bioluminescence imaging showed significantly less whole body and head parasite accumulation in CD8^−/−^ mice when compared to WT or CD4^−/−^ mice between days 7 and 9, at the time when WT mice started to develop ECM (Figure 2C and D). Further quantification of IRBC accumulation in isolated organs was performed on day 7. We observed significantly decreased IRBC accumulation in the brain (Figures 2A and 2F and S2A) and the spleen (Figure 2E) for CD8^−/−^ mice but not in the other organs tested (Figure S3A to D). IRBC accumulation in the tested organs of CD4^−/−^ mice was similar to that of WT mice except for the spleen, where accumulation was reduced (Figure S3A to D).

**Figure 3:**
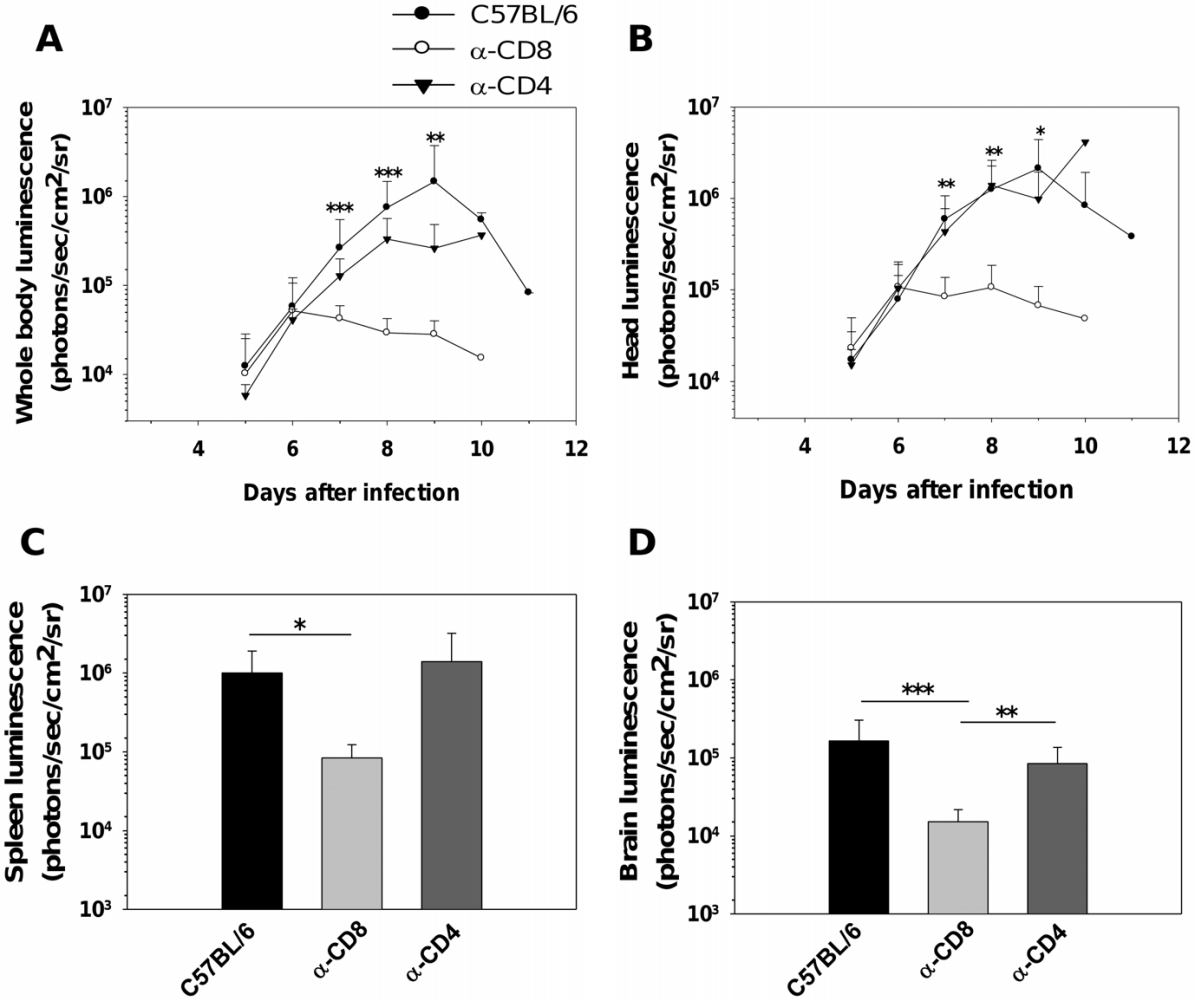
Decrease in parasite biomass and IRBC accumulation in organs from CD8^+^ T cell-depleted WT mice. *In vivo* bioluminescence imaging quantification of IRBC accumulation in the whole body (A) and head (B) of mice infected with PbA*luc* and depleted of either CD4^+^ or CD8^+^ T cells by antibody treatment. *Ex vivo* quantification by bioluminescence imaging of IRBC accumulation in spleens (C) and brains (D). Data are expressed as mean ± SD. In this experiment, all the control (*n* = 10) and CD4^+^-depleted (*n* = 5) mice infected with PbA*luc* developed ECM between days 7 and 12, and none of the anti-CD8^+^-treated (*n* = 10) infected mice developed CM. On each day that a control-infected mouse developed CM, one CD4 depleted- and one CD8-depleted mouse were sacrificed to determine IRBC accumulation *ex vivo*. Luminescence values (log) as mean ± SD of 5 mice. *p<0.05, **p<0.01 and ***p<0.001, ANOVA followed by Bonferroni test.

Previous studies have shown that depletion of CD8^+^ but not CD4^+^ T cells 6 days post-infection, just prior to the onset of neurological signs, prevents the development of ECM [22]. Thus, we applied these treatments to PbA*luc*-infected mice and measured IRBC accumulation when untreated mice developed clinical signs of ECM. None (out of 5) of the infected mice depleted with anti-CD8 developed ECM. On the contrary, most mice (3 out of 5) depleted with anti-CD4 mAbs developed ECM, to the same extent as WT C57BL/6 mice (7 out of 8 mice). Parasitemia developed similarly in control groups and in CD4^+^- and CD8^+^-depleted groups (data not shown). This confirmed previous results [22]. In agreement with the data above using deficient mice, significantly fewer IRBC were accumulated in the bodies and heads of CD8^+^-depleted mice than in WT or CD4^+^ -depleted mice (Figure 3A and 3B). Fewer IRBC were detected in isolated brains (Figure 3D and 2B), spleens (Figure 3C) and in other organs tested (liver, lungs, kidneys and heart) of CD8^+^-depleted mice as compared to WT or CD4^+^-depleted mice (Figures S2B and S3E to H). Although IRBC accumulation in the brain and spleen were similar for CD8^−/−^ and CD8-depleted mice, the absence of differences in other organs tested from CD8^−/−^ mice suggest that compensatory mechanisms that lead to parasite accumulation may develop in these mice. Taken together, our results show that CD8^+^ but not CD4^+^ T cells are the main cellular subset controlling IRBC accumulation in the brain and organs. They also demonstrate that accumulation of IRBC per se is not sufficient for ECM to occur.

### Myeloid cells are not involved in IRBC accumulation in the brain and other organs

**Figure 4:**
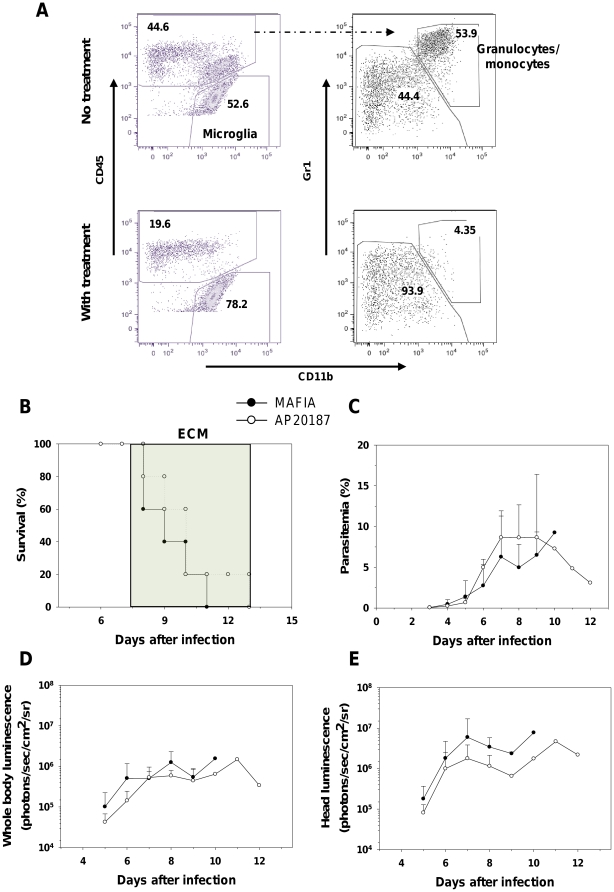
Depletion of myeloid cells does not alter IRBC distribution during ECM. MAFIA mice were infected with PbA*luc* and injected on days 5, 6, and 7 with the drug AP20187 as described in Material and Methods. (A) Depletion of granulocytes/monocytes (defined as CD45^+^CD11b^+^Gr1^+^) was assessed by flow cytometry on day 7 post-infection. Data plots presented are from one mouse and similar data were obtained for 4 more mice. (B) Survival and (C) parasitemia of treated and non-treated MAFIA mice (5 per group). Neurologic signs of CM appeared on days 6–12 (shaded area), with death occurring 24–48 h after onset. Parasitemia (%) values are expressed as mean ± SD of 5 mice per group. *In vivo* bioluminescence imaging quantification of IRBC accumulation in the whole body (D) and head (E) of treated and non-treated infected mice. Luminescence values (log) as mean ± SD of 5 mice.

Myeloid cells (both neutrophils and monocytes) have been shown to accumulate at the time of ECM in the brains of infected animals [22]. Since these cells have the capacity to phagocytose IRBC [42], [43] and also to adhere to the activated endothelium, we tested the possibility that increased bioluminescence signals in organs may in fact be due to sequestration of myeloid cells that have ingested IRBC. To do so, we took advantage of a new transgenic mouse model, MAFIA, expressing an inducible suicide gene under the promoter of the M-CSFR (CD115) promoter allowing conditional ablation of myeloid CD115^+^ cells after treatment with the dimerizer cytotoxic peptide AP20187 [33]. MAFIA mice were infected with PbA*luc* and treated (or not) with the dimerizer peptide. At day 8 post-infection, granulocyte/monocyte depletion was about 80% complete in the blood (Figure S4), the brain (Figure 4A), and in the lungs (data not shown) of treated MAFIA mice as determined by flow cytometry. Depletion of myeloid cells did not prevent ECM (Figure 4B) or modify parasitemia (Figure 4C). This treatment also did not affect parasite biomass for the whole body (Figure 4D) or the head (Figure 4E). Additional analysis on isolated organs confirmed these findings (Figure S5). We also tested the role of myeloid cells using CCR2^−/−^ mice. These mice deficient for a chemokine receptor are fully susceptible to ECM but have a strong reduction in monocyte (50%) and neutrophil (100%) numbers in their brains at the time of ECM when compared to WT mice [44]. To further prevent residual monocyte migration to the brain during ECM, we treated the mice with an anti-mouse M-CSF receptor mAb (AFS98) which prevents monocyte migration into tissues without depleting them [38], [45]. CCR2^−/−^ mice treated at day 5 and 6 post-infection still die of ECM and have the same parasitemia as CCR2^−/−^ non-treated mice (Figure S6A and S6B). There were also no significant differences in parasite biomass in the whole body and in the head between antibody-treated and untreated mice (Figure S6C and S6D). Our results clearly show that monocytes and neutrophils are not involved in IRBC accumulation and confirmed that these cells are not involved in ECM.

### Cytokine control of IRBC accumulation in organs

**Figure 5:**
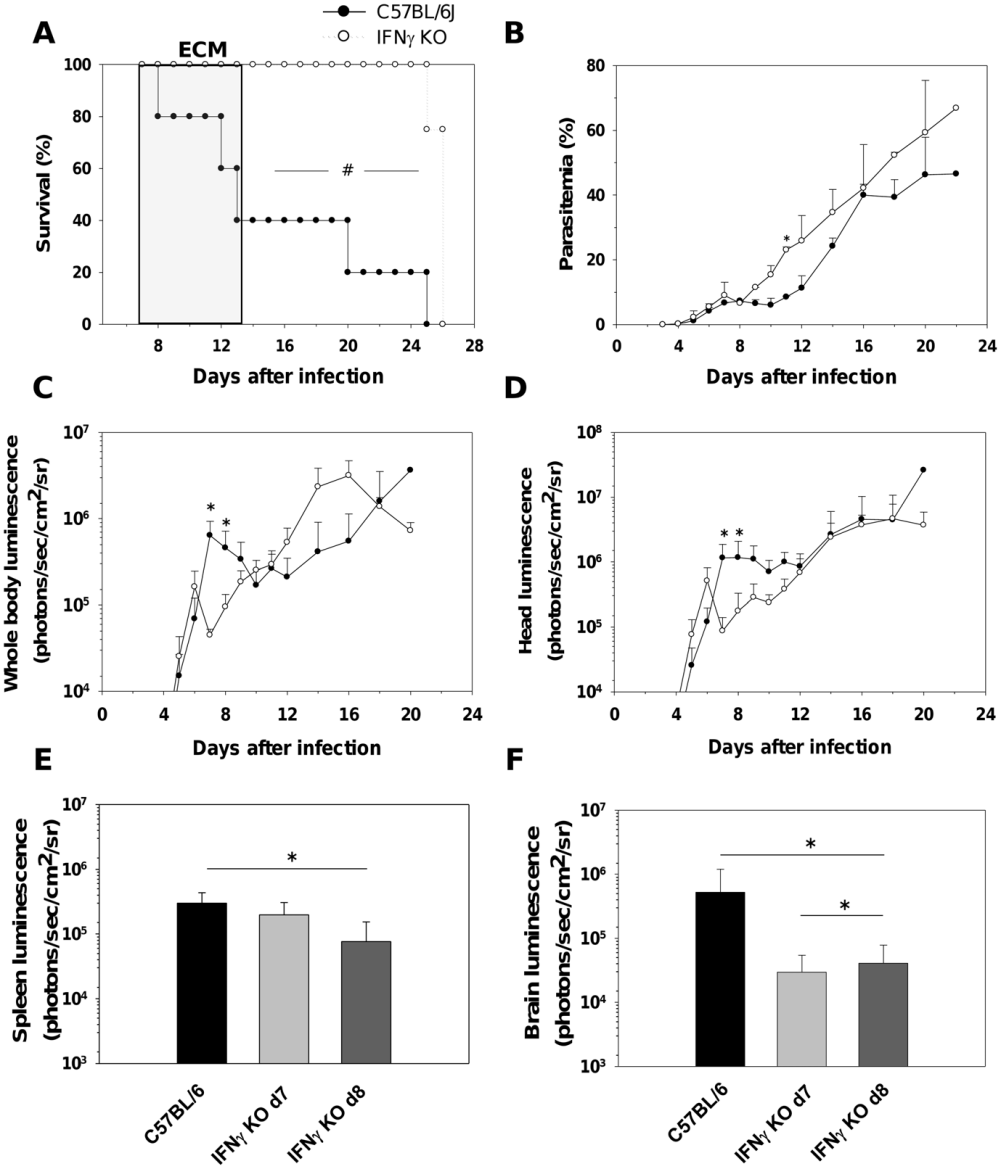
IFN-γ controls parasite biomass and IRBC accumulation in organs. (A) Survival and (B) parasitemia of WT and IFN-γ^−/−^ mice (5 mice per group) infected with PbA*luc*. Parasitemia (%) values are expressed as mean ± SD. *In vivo* bioluminescence imaging quantification of IRBC accumulation in the whole body (C) and head (D) of WT and IFN-**γ** ^−/−^ mice. Luminescence values (log) as mean ± SD of 5 mice. *Ex vivo* quantification by bioluminescence imaging of IRBC accumulation in spleens (E) and brains (F) of perfused WT and IFN-γ^−/−^ mice obtained at day 7 and 8 post-infections. Luminescence values (log) as mean ± SD of 5 mice. ^#^p<0.01, log-rank test; *p<0.05, Mann Whitney test; *p<0.05, ANOVA followed by Bonferroni test.

Proinflammatory cytokines, such as IFN-γ [24] and lymphotoxin-α [25] play important roles in the pathogenesis of ECM in part by inducing CD8^+^ T cell migration to the brain and other organs [46]. However, their impact in IRBC accumulation is unknown. We thus infected IFN-γ^−/−^ mice with PbA*luc*; these mice were completely resistant to ECM (Figure 5A) despite developing parasitemia levels similar to or higher than WT (Figure 5B). Bioluminescence imaging showed that IFN-γ^−/−^ mice had lower total or head parasite biomass than the WT mice, but remarkably only during the time frame when the latter developed ECM (Figure 5C and 5D). IRBC accumulation was assessed in isolated organs on days 7 and 8 post-infection (at the time when WT mice started to develop ECM). Significantly fewer IRBC were accumulated in the brains of IFN-γ^−/−^ mice than in WT mice on both days (Figures 5F and S7). In the other organs, there was no statistically significant difference in IRBC accumulation (Figure S8) except for the spleen, where the IFN-γ^−/−^ mice showed a modest decrease in parasite biomass on day 8 (Figure 5E). The time- and location-specific effect of IFN-γ on parasite accumulation strongly suggests a causative role for this cytokine in ECM pathogenesis.

We also tested the role of two other pro-inflammatory cytokines, namely TNF-α and IL-12 using deficient mice. Both types of mice were fully susceptible to ECM and their parasitemias did not differ from those of WT mice. In addition, absence of these cytokines did not influence parasite accumulation in the whole body or isolated organs during infection (Figures S9, S10 and S11).

## Discussion

Pathologies induced by the malaria infection result from the complex interplay between the parasite and the immune system [6]. In particular, it has been proposed that IRBC sequestration, resulting from the cytoadherence of IRBC to endothelium and local inflammation, is the principal cause of pathogenesis [47]. Moreover, high *P. falciparum* parasite biomass representing both circulating and sequestered parasites has been shown to be associated with severity of the infection in humans [48]. In this article, we have investigated the influence of immune cells and cytokines of IRBC accumulation in various organs during infection with PbA, an experimental model of CM.

We and others have previously reported that T cells, both CD4^+^ and CD8^+^, are essential for ECM to develop [22], [41], [49], [50], CD4^+^ T cells being involved in the induction phase and CD8^+^ T cells in the effector phase [22]. However their role in parasite growth and distribution has not been clearly defined. In this study, we first observed that RAG2^−/−^ mice which lack T and B cells had a reduced total parasite biomass and accumulated fewer parasites in their brains during the first week of the infection but not at later time points. Since these mice still have NK cells, this rules out a direct role for these cells in IRBC accumulation or distribution in tissues. Using deficient mice and antibody mediated-depletion, we further showed that CD8^+^ but not CD4^+^ T cells mediate the growth in total and head parasite biomass coinciding with ECM development. *In vivo* parasite biomass measurements in the head mirrored the reduction of IRBC accumulation in the brains isolated from CD8^−/−^ mice infected 7 days earlier (Figure 2F) and from mice treated with anti-CD8^+^ antibodies at day 6 post-infection, just before ECM onset (Figure 3D). CD8^+^ T cells have previously been reported to be sequestered in the brain [21] when PbA-infected mice displayed overt ECM signs, which is also when we observed a surge in IRBC accumulation. We recently observed that C57BL/6J ECM-susceptible mice have a higher parasite load in the brain than ECM-resistant BALB/cJ mouse strain at the time of ECM signs (Claser and Renia, unpublished). In the resistant mouse strain, CD8^+^ T cells also do not accumulate in the brain of infected mice [22]. Taken together, this suggests that brain-sequestered CD8^+^ T cells are responsible for the time-dependent accumulation of PbA IRBC.

How can CD8^+^ T cells influence IRBC accumulation in tissues? Although there is ample evidence that IRBC do accumulate in tissues, the mechanism of accumulation is unknown [26]–[28]. In humans infected with *P. falciparum*, sequestration of IRBC in deep tissue results from their cytoadherence to endothelial cells [19]. However, this phenomenon has yet to be demonstrated *in vivo* or *in vitro* for PbA. Different scenarios can be proposed to explain our results. If PbA IRBC do cytoadhere, then it is possible that CD8^+^ T cells, through direct interaction and/or release of mediators, activate endothelial cells and favor binding of IRBC. We previously proposed that binding of IRBC to activated cytokines would leads to IRBC phagocytosis and processing and presentation to CD8+ T cells [51]. This would explain the implication of perforin in ECM [50], [52].

If PbA IRBC do not cytoadhere, then their accumulation may result from hemodynamic disturbances or microhemorrhages. One possible mechanism is the blockade of capillaries by other cells also sequestered intravascularly. Monocytes and neutrophils are the principal cell subsets sequestered in brain capillaries during ECM [22]. They also have the capacity to phagocytose IRBC and thus may carry undigested parasites. We used two experimental systems (MAFIA mice and CCR2^−/−^ mice treated with a migration-blocking antibody) that allow significant reduction in the numbers of these cell subsets in deep tissues. In both systems, no modification of IRBC accumulation was observed (Figures 4 and S5). In addition, infected RAG2^−/−^ mice that possess normal monocyte and neutrophil compartments did accumulate fewer IRBC than WT mice (Figures 1 and S2). This clearly demonstrates that these two cell subsets were not responsible for IRBC accumulation and that the CD8^+^ T cell effect was not mediated by these cells. We then tested the role of pro-inflammatory cytokines, which have been shown to activate endothelial cells during ECM [17]. We demonstrated that IFN-γ, but not TNF-α and IL-12, is involved in the control of IRBC accumulation in tissues. Moreover, the effect of IFN-γ was tissue-specific, manifesting in the brain and to a lesser extent, the spleen, but not in the other organs tested (Figures 5E, 5F and S6).

During completion of this work, two studies also reported a role for CD8^+^ T cells in controlling IRBC accumulation in the brains [32] and other organs [32] of PbA-infected mice, thus confirming our results. It is not known if IRBC accumulation during PbA infection in deep tissues leads to organ-specific pathologies as has been proposed for *P. falciparum* malaria [48]. However, it has been reported that CD8^+^ T cells induce damage such as vascular permeability in the lungs and kidneys during PbA infection in C57BL/6J mice [21]. By extension to the brain, it is thus possible that CD8^+^-mediated IRBC accumulation may be responsible for the various organ pathologies seen during ECM. Surprisingly, although infected CD8^−/−^ mice showed the same reduction in IRCB accumulation in the spleen and brain as CD8-depleted mice, there was no difference in other organs such as the heart, lung, liver and kidneys at 7 to 10 days post-infection (Figures 2 and S2). A possible explanation is the development of organ-specific compensatory mechanisms in these mice since CD8^+^ T cells were absent from the start of the infection.

In our hands, PbA-infected-CD4^−/−^ mice did not show much modification of IRBC accumulation in tissues except for the spleen as compared to WT mice. This contrasts with data reported very recently by Amante *et al.* [32]. In this study, CD4^+^ T cell depletion was performed using antibody before and after infection and was shown to modify parasite biomass to the same extent as CD8^+^ T cell depletion. We also observed differences for TNF-α between the two studies. Amante *et al.* [32] found that a higher parasite biomass in infected TNF-α KO mice. In our hands, TNF-α has no effect on parasite accumulation in tissues (Figure S9). Quantitative or qualitative differences in the parasite used or the route of inoculation may account for the discrepancy between the two studies, but more studies are needed to clarify this issue.

In conclusion, we have shown that CD8^+^ T cells and IFN-γ mediate IRBC accumulation in the brain and in other organs during ECM at the time when mice develop clinical signs. Lymphotoxin-α has been shown by others also to be involved in this pathogenic process [32] and essential for ECM to occur [25], [53]. This reveals a complex interplay between the parasite and the immune system, defying an either-or choice between the mechanical and immunological hypotheses of CM and supporting an integrated mechanism. Our data open the way to new testable hypotheses for the pathogenesis of human CM.

## Supporting Information

Figure S1 **Positioning of mice during measurement of bioluminescence.** Mice were injected with luciferin and placed in a (A) ventral position for whole body imaging and (B) dorsal position for head imaging.
(TIFF)

Figure S2 **Reduced IRBC accumulation in the brains of CD8^−/−^ mice or CD8^+^ T cell-depleted mice.** Pseudocolor images of isolated brains from perfused (A) CD4^−/−^, CD8^−/−^, and WT or (B) anti-CD4^+^-treated, anti-CD8^+^-treated and untreated mice.
(TIFF)

Figure S3 **Reduced IRBC accumulation in different organs of CD8^+^ T cell-depleted mice.** Quantification of IRBC accumulation was performed in CD4^−/−^, CD8^−/−^, and WT (A–D) or anti-CD4^+^-treated, anti-CD8^+^-treated and untreated (E–H) mice infected 7 days before. Mice were injected with luciferin and IRBC accumulation was measured by bioluminescence imaging in organs obtained from perfused mice as described in Materials and Methods. Luminescence values (log) as mean ± SD of 5 mice per group. **p<0.01 and ***p<0.001, ANOVA followed by Bonferroni post-test.
(TIFF)

Figure S4 **Efficacy of granulocyte/monocyte depletion in peripheral blood of MAFIA mice after treatment with the drug AP20187.** Mice were infected with PbA*luc* and injected on day 5, 6, and 7 with the drug AP20187 as described in Material and Methods. Depletion of granulocytes/monocytes (defined as CD45^+^CD11b^+^Gr1^+^) was assessed by flow cytometry on day 7 post-infection. Data plots presented are from one mouse and similar data were obtained for 4 more mice.
(TIFF)

Figure S5 **Depletion of myeloid cells does not reduce IRBC accumulation in different organs of infected MAFIA mice.** MAFIA mice were infected with PbA*luc* and injected on days 5, 6, and 7 with the drug AP20187 as described in Material and Methods. (A–F) *Ex vivo* quantification by bioluminescence imaging of IRBC accumulation in organs obtained from perfused mice treated or not treated with the drug AP20187 at day 7 post-infection. Luminescence values (log) as mean ± SD of 5 mice per group.
(TIFF)

Figure S6 **Monocytes and neutrophils are not involved in IRBC accumulation.** (A) Survival and (B) parasitemia of WT, anti-M-CSF-R antibody-treated and control CCR2^−/−^ mice infected with PbA*luc*. CCR2^−/−^ mice received 2 mg of anti-M-CSF-R (AFS98) antibody on days 5 and 6 post-infection. Neurologic signs of CM appeared on days 7–10 (shaded area), with death occurring 24–48 h after onset. Parasitemia (%) values are expressed as mean ± SD of 5 mice per group. *In vivo* bioluminescence imaging quantification of IRBC accumulation in the whole body (C) and head (D) of infected mice. Luminescence values (log) as mean ± SD of 5 mice per group.
(TIFF)

Figure S7 **Reduced IRBC accumulation in the brains of IFN-γ^−/−^ mice.** Pseudocolor images of isolated brains from perfused WT and IFN-γ^−/−^ mice at day 7 post-infection.
(TIFF)

Figure S8 **IFN-γ deficiency does not influence IRBC accumulation in different organs.** (A–D) IRBC accumulation was determined by bioluminescence imaging of isolated organs from perfused WT and IFN-γ^−/−^ mice infected with PbA*luc* at days 7 and 8 post-infection. Luminescence values (log) as mean ± SD of 5 mice per group.
(TIFF)

Figure S9 **TNF-α is not involved in IRBC accumulation in the whole body and head.** (A) Survival and (B) parasitemia of WT and TNFα^−/−^ mice infected with PbAluc. Neurologic signs of CM appeared on days 6–12 (shaded area), with death occurring 24–48 h after onset. Data are expressed as mean ± SD of 5 mice per group. Parasitemia (%) values are expressed as mean ± SD of 5 mice per group. In vivo bioluminescence imaging quantification of IRBC accumulation in the whole body (C) and head (D) of infected mice. Data are expressed as mean ± SD of 5 mice per group. Luminescence values (log) as mean ± SD of 5 mice per group.
(TIFF)

Figure S10 **IL12 is not involved in IRBC accumulation in the whole body and head.** (A) Survival and (B) parasitemia of WT (*n* = 10) and IL12p40^−/−^ (*n* = 19) mice infected with PbA*luc*. Neurologic signs of CM appeared on days 6–12 (shaded area), with death occurring 24–48 h after onset. In WT mice, neurological signs appear on day 8–12 (shaded area). Parasitemia (%) values are expressed as mean ± SD. *In vivo* bioluminescence imaging quantification of IRBC accumulation in the whole body (C) and head (D) of infected mice. Luminescence values (log) as mean ± SD of 5 mice.
(TIFF)

Figure S11 **TNF-α and IL12 are not involved in IRBC accumulation in organs.** (A–F) Quantification of IRBC accumulation was performed in organs from perfused TNF-α ^−/−^, IL12p40^−/−^ mice and WT mice infected with PbA*luc* parasites 7 days before. Luminescence values (log) as mean ± SD of 5 mice per group.
(TIFF)

## References

[1] Feachem RG, Phillips AA, Hwang J, Cotter C, Wielgosz B (2010). Shrinking the malaria map: progress and prospects.. Lancet.

[2] Hay SI, Okiro EA, Gething PW, Patil AP, Tatem AJ (2010). Estimating the global clinical burden of *Plasmodium falciparum* Malaria in 2007.. Plos Med.

[3] Severe falciparum malaria. World Health Organization, Communicable Diseases Cluster (2000). Trans R Soc Trop Med Hyg.

[4] van der Heyde HC, Nolan J, Combes V, Gramaglia I, Grau GE (2006). A unified hypothesis for the genesis of cerebral malaria: sequestration, inflammation and hemostasis leading to microcirculatory dysfunction.. Trends Parasitol.

[5] Idro R, Jenkins NE, Newton CRJC (2005). Pathogenesis, clinical features, and neurological outcome of cerebral malaria.. Lancet Neurol.

[6] Schofield L, Grau GE (2005). Immunological processes in malaria pathogenesis.. Nat Rev Immunol.

[7] Berendt AR, Turner GDH, Newbold CI (1994). Cerebral malaria: the sequestration hypothesis.. Parasitol Today.

[8] Miller LH (1969). Distribution of mature trophozoites and schizonts of *Plasmodium falciparum* in the organs of *Aotus trivirgatus*, the night monkey.. Am J Trop Med Hyg.

[9] Bignami A, Bastianelli G (1889). Observations on aestivo-autumnal malarial fevers.. Riforma Med.

[10] MacPherson GG, Warrell MJ, White NJ, Looareesuwan S, Warrell DA (1985). Human cerebral malaria. A quantitative ultrastructural analysis of parasitized erythrocyte sequestration.. Am J Pathol.

[11] Ewing J (1901). Contribution of the pathological anatomy of malaria fever.. J Exp Med.

[12] Taylor TE, Fu WJ, Carr RA, Whitten RO, Mueller JG (2004). Differentiating the pathologies of cerebral malaria by postmortem parasite counts.. Nat Med.

[13] Grau GE, Taylor TE, Molyneux ME, Wirima JJ, Vassalli P (1989). Tumor necrosis factor and disease severity in children with *falciparum* Malaria.. N Engl J Med.

[14] Kwiatkowski DP, Hill AVS, Sambou I, Twumasi PM, Castracane J (1990). TNF concentration in fatal cerebral, non fatal cerebral, and uncomplicated *Plasmodium falciparum* malaria.. Lancet.

[15] Patnaik JK, Das BS, Mishra SK, Mohanty S, Satpathy SK (1994). Vascular clogging, mononuclear cell margination, and enhanced vascular permeability in the pathogenesis of human cerebral malaria.. Am J Trop Med Hyg.

[16] Grau GE, Mackenzie CD, Carr RA, Redard M, Pizzolato G (2003). Platelet accumulation in brain microvessels in fatal pediatric cerebral malaria.. J Infect Dis.

[17] Hunt NH, Grau GE (2003). Cytokines: accelerators and brakes in the pathogenesis of cerebral malaria.. Trends Immunol.

[18] Berendt AR, Simmons DL, Tansey J, Newbold CI, Marsh K (1989). Intercellular Adhesion Molecule-1 Is an endothelial cell adhesion receptor for *Plasmodium falciparum*.. Nature.

[19] Chen Q, Schlichtherle M, Wahlgren M (2000). Molecular aspects of severe malaria.. Clin Microbiol Rev.

[20] Engwerda CR, Belnoue E, Gruner AC, Renia L (2005). Experimental models of cerebral malaria.. Curr Top Immunol.

[21] Chang WL, Jones SP, Lefer DJ, Welbourne T, Sun G (2001). CD8^+^-T-Cell Depletion ameliorates circulatory shock in *Plasmodium berghei*-infected mice.. Infect Immun.

[22] Belnoue E, Kayibanda M, Vigario AM, Deschemin JC, van Rooijen N (2002). On the pathogenic role of brain-sequestered alpha beta CD8^+^ T cells in experimental cerebral malaria.. J Immunol.

[23] Hansen DS, Bernard NJ, Nie CQ, Schofield L (2007). NK Cells Stimulate Recruitment of CXCR3+ T Cells to the brain during *Plasmodium berghei*-Mediated Cerebral Malaria.. J Immunol.

[24] Amani V, Vigario AM, Belnoue E, Marussig M, Fonseca L (2000). Involvement of IFN-gamma receptor-mediated signaling in pathology and anti-malarial immunity induced by *Plasmodium berghei* infection.. Eur J Immunol.

[25] Engwerda CR, Mynott TL, Sawhney S, De Souza JB, Bickle QD (2002). Locally Up-regulated Lymphotoxin alpha, not systemic Tumor Necrosis Factor alpha, is the principle mediator of murine cerebral malaria.. J Exp Med.

[26] Hulier E, Petour P, Snounou G, Nivez MP, Miltgen F (1996). A method for the quantitative assessment of malaria parasite development in organs of the mammalian host.. Mol Biochem Parasitol.

[27] Jennings VM, Actor JK, Lal AA, Hunter RL (1997). Cytokine profile suggesting that murine cerebral malaria is an encephalitis.. Infect Immun.

[28] Hearn J, Rayment N, Landon DN, Katz DR, De Souza JB (2000). Immunopathology of Cerebral Malaria: morphological evidence of parasite sequestration in murine brain microvasculature.. Infect Immun.

[29] Franke-Fayard BM, Fonager J, Braks A, Khan SM (2010). Sequestration and tissue accumulation of human malaria parasites: can we learn anything from rodent models of malaria?. PLoS Pathog.

[30] Franke-Fayard BM, Janse CJ, Cunha-Rodrigues M, Ramesar J, Buscher P (2005). Murine malaria parasite sequestration: CD36 is the major receptor, but cerebral pathology is unlinked to sequestration.. Proc Natl Acad Sci USA.

[31] Amante FH, Stanley AC, Randall LM, Zhou Y, Haque A (2007). A Role for Natural regulatory T cells in the pathogenesis of experimental cerebral malaria.. Am J Pathol.

[32] Amante FH, Haque A, Stanley AC, de Labastida Rivera F, Randall LM (2010). Immune-mediated mechanisms of parasite tissue sequestration during experimental cerebral malaria.. J Immunol.

[33] Burnett SH, Kershen EJ, Zhang J, Zeng L, Straley SC (2004). Conditional macrophage ablation in transgenic mice expressing a Fas-based suicide gene.. J Leukoc Biol.

[34] Amani V, Boubou MI, Pied S, Marussig M, Walliker D (1998). Cloned lines of *Plasmodium berghei* ANKA differ in their abilities to induce experimental cerebral malaria.. Infect Immun.

[35] Belnoue E, Voza T, Costa FTM, Gruner AC, Mauduit M (2008). Vaccination with Live *Plasmodium yoelii* Blood stage parasites under chloroquine cover Induces cross-stage immunity against malaria liver Stage.. J Immunol.

[36] Sudo T, Nishikawa S, Ogawa M, Kataoka H, Ohno N (1995). Functional hierarchy of c-kit and c-fms in intramarrow production of CFU-M.. Oncogene.

[37] Jose MD, Le Meur Y, Atkins RC, Chadban SJ (2003). Blockade of macrophage colony-stimulating factor reduces macrophage proliferation and accumulation in renal allograft rejection.. Am J Transplant.

[38] Murayama T, Yokode M, Kataoka H, Imabayashi T, Yoshida H (1999). Intraperitoneal administration of anti-c-fms monoclonal antibody prevents initial events of atherogenesis but does not reduce the size of advanced lesions in apolipoprotein E-deficient mice.. Circulation.

[39] Brosnan CF, Shafit-Zagardo B, Aquino DA, Berman JW (1993). Expression of monocyte/macrophage growth factors and receptors in the central nervous system.. Adv Neurol.

[40] Wang Y, Berezovska O, Fedoroff S (1999). Expression of colony stimulating factor-1 receptor (CSF-1R) by CNS neurons in mice.. J Neurosci Res.

[41] Yanez DM, Manning DD, Cooley AJ, Weidanz WP, van der Heyde HC (1996). Participation of lymphocyte subpopulations in the pathogenesis of experimental murine cerebral malaria.. J Immunol.

[42] Schetters TPM, van Run-van Breda JH, Eling WMC (1983). Immune phagocytosis of *Plasmodium berghei* parasites by mononuclear phagocytes.. Cell Biol Int Rep.

[43] Celada A, Cruchaud A, Perrin LH (1983). Phagocytosis of *Plasmodium falciparum*-parasitized erythrocytes by human polymorphonuclear leukocytes.. J Parasitol.

[44] Belnoue E, Costa FTM, Vigario AM, Voza T, Gonnet F (2003). Chemokine receptor CCR2 is not essential for the development of experimental cerebral malaria.. Infect Immun.

[45] Segawa M, Fukada S, Yamamoto Y, Yahagi H, Kanematsu M (2008). Suppression of macrophage functions impairs skeletal muscle regeneration with severe fibrosis.. Exp Cell Res.

[46] Belnoue E, Potter SM, Rosa DS, Mauduit M, Gruner AC (2008). Control of pathogenic CD8+ T cell migration to the brain by IFN-gamma during experimental cerebral malaria.. Parasite Immunol.

[47] Miller LH, Baruch DI, Marsh K, Doumbo OK (2002). The pathogenic basis of malaria.. Nature.

[48] Dondorp AM, Desakorn V, Pongtavornpinyo W, Sahassananda D, Silamut K (2005). Estimation of the total parasite biomass in acute falciparum malaria from plasma PfHRP2.. Plos Med.

[49] Bagot S, Nogueira F, Collette A, Do Rosario VE, Lemonier F (2004). Comparative study of brain CD8+ T cells Induced by sporozoites and those Induced by blood-Stage *Plasmodium berghei* ANKA involved in the development of cerebral malaria.. Infect Immun.

[50] Nitcheu J, Bonduelle O, Combadiere C, Tefit M, Seilhean D (2003). Perforin-Dependent brain-infiltrating cytotoxic CD8+ T lymphocytes mediate experimental cerebral malaria pathogenesis.. J Immunol.

[51] Renia L, Potter SM, Mauduit M, Rosa DS, Kayibanda M (2006). Pathogenic T cells in cerebral malaria.. Int J Parasitol.

[52] Potter SM, Chan-Ling T, Ball HJ, Mansour H, Mitchell AJ (2006). Perforin mediated apoptosis of cerebral microvascular endothelial cells during experimental cerebral malaria.. Int J Parasitol.

[53] Togbe D, de Sousa PL, Fauconnier M, Boissay V, Fick L (2008). Both functional LTbeta receptor and TNF receptor 2 are required for the development of experimental cerebral malaria.. PLoS ONE.

