# Supplementary figures and images for: CD8+ T Cells and IFN-γ Mediate the Time-Dependent Accumulation of Infected Red Blood Cells in Deep Organs during Experimental Cerebral Malaria
Source: PLoS One. 2011 Apr 11;6(4):e18720. doi: 10.1371/journal.pone.0018720 (PMC3073989; doi:10.1371/journal.pone.0018720)

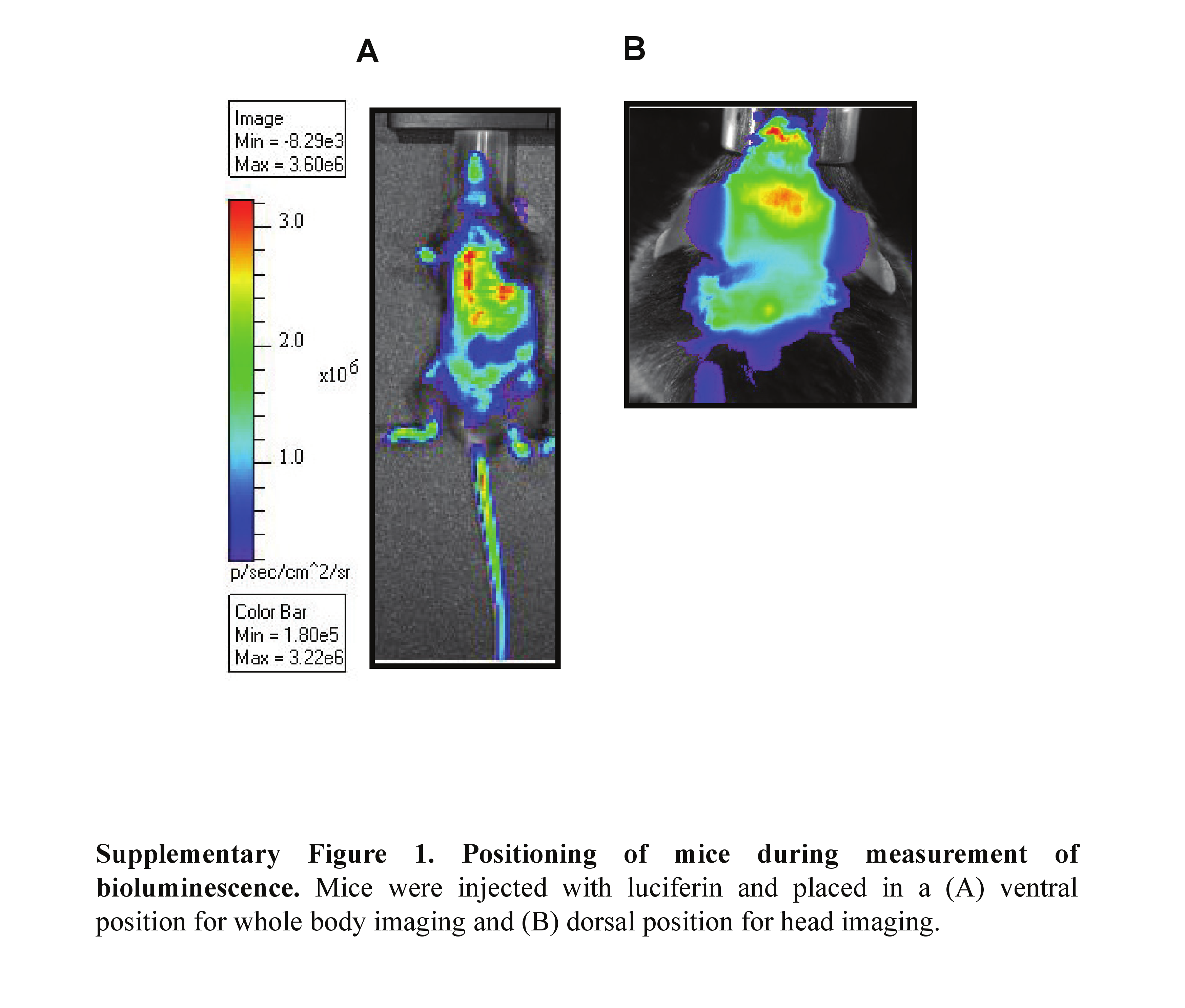

Supplement: Figure S1 — Positioning of mice during measurement of bioluminescence. Mice were injected with luciferin and placed in a (A) ventral position for whole body imaging and (B) dorsal position for head imaging. (TIFF) [file pone.0018720.s001.tif]

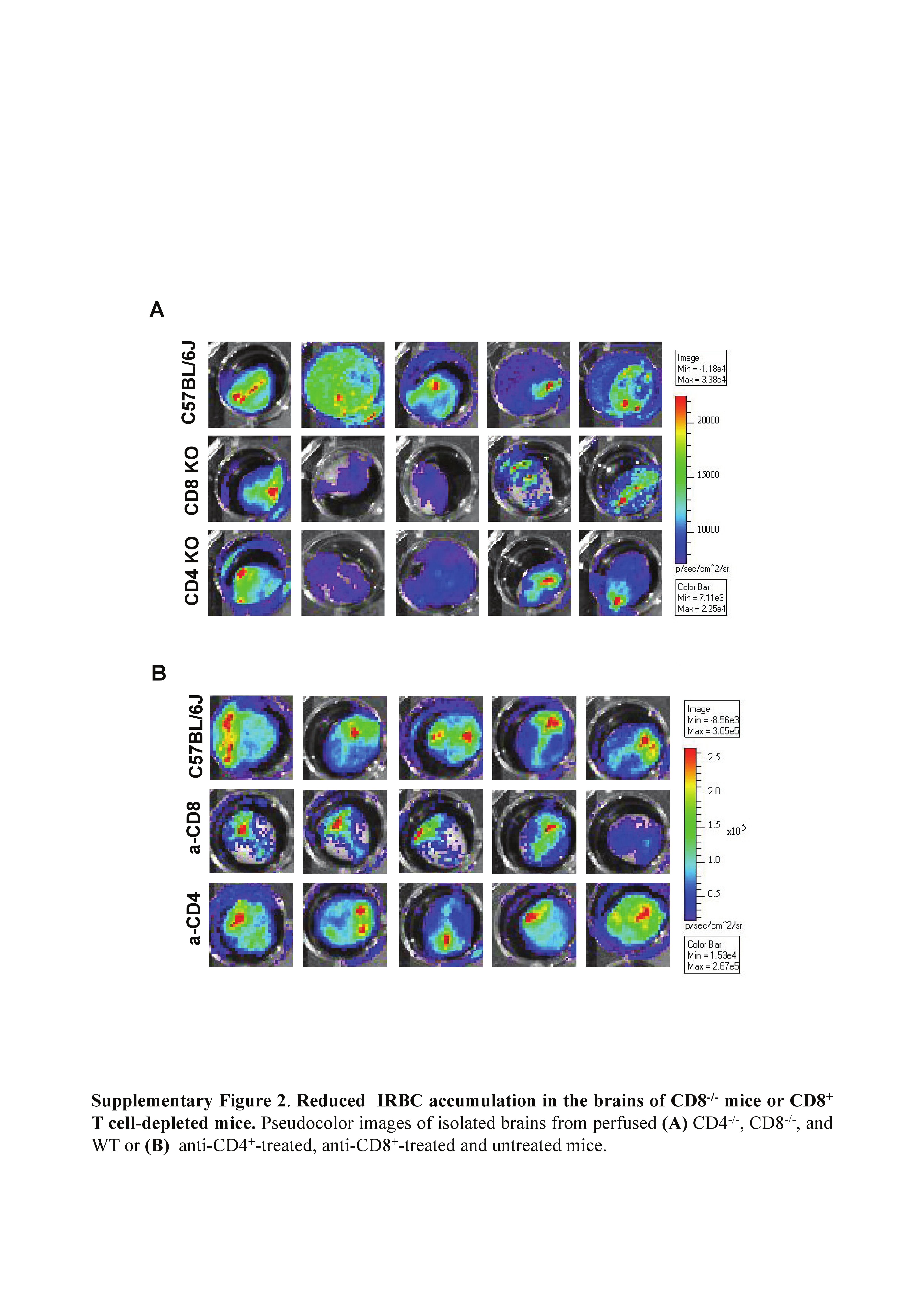

Supplement: Figure S2 — Reduced IRBC accumulation in the brains of CD8−/− mice or CD8+ T cell-depleted mice. Pseudocolor images of isolated brains from perfused (A) CD4−/−, CD8−/−, and WT or (B) anti-CD4+-treated, anti-CD8+-treated and untreated mice. (TIFF) [file pone.0018720.s002.tif]

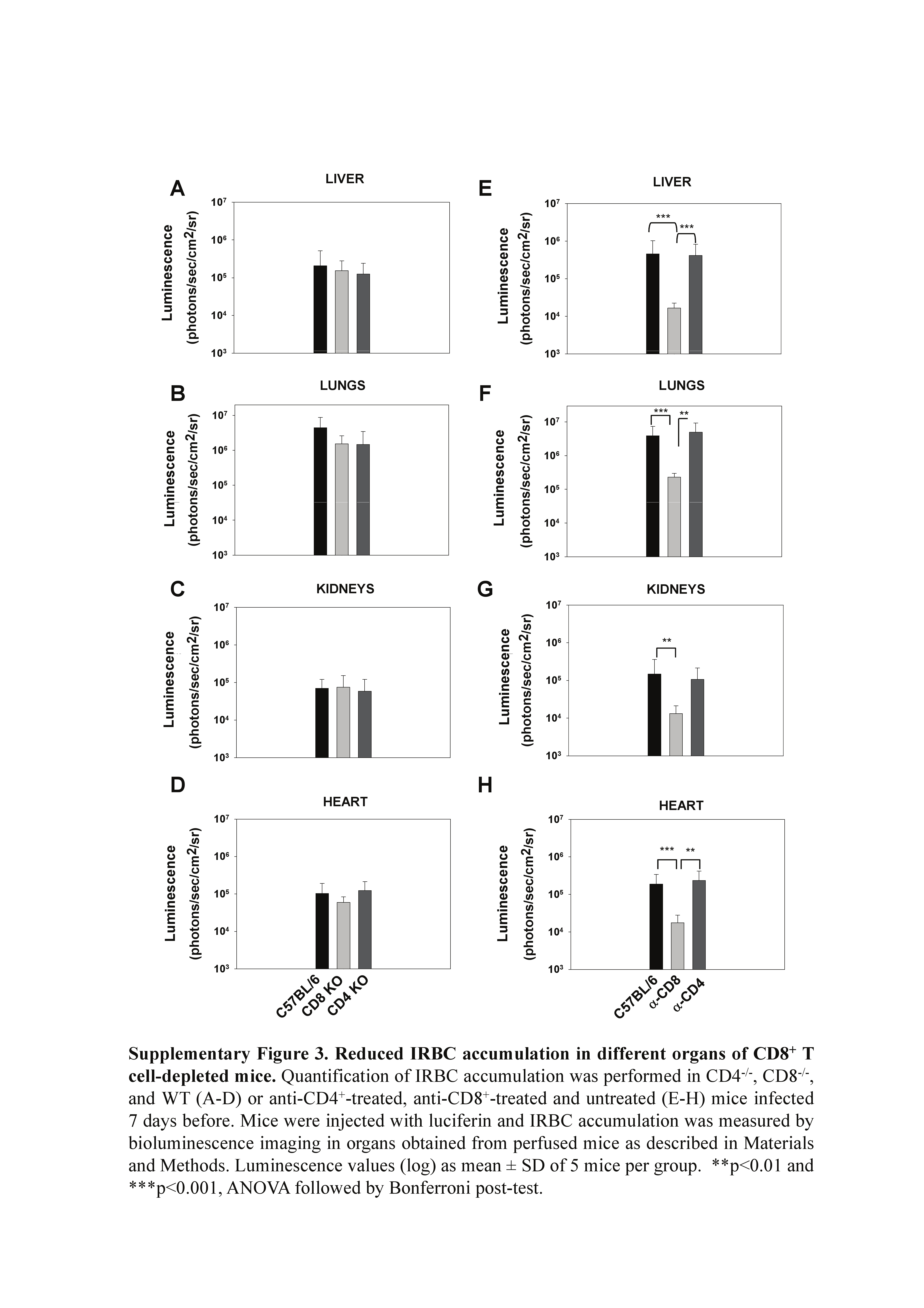

Supplement: Figure S3 — Reduced IRBC accumulation in different organs of CD8+ T cell-depleted mice. Quantification of IRBC accumulation was performed in CD4−/−, CD8−/−, and WT (A–D) or anti-CD4+-treated, anti-CD8+-treated and untreated (E–H) mice infected 7 days before. Mice were injected with luciferin and IRBC accumulation was measured by bioluminescence imaging in organs obtained from perfused mice as described in Materials and Methods. Luminescence values (log) as mean ± SD of 5 mice per group. **p<0.01 and ***p<0.001, ANOVA followed by Bonferroni post-test. (TIFF) [file pone.0018720.s003.tif]

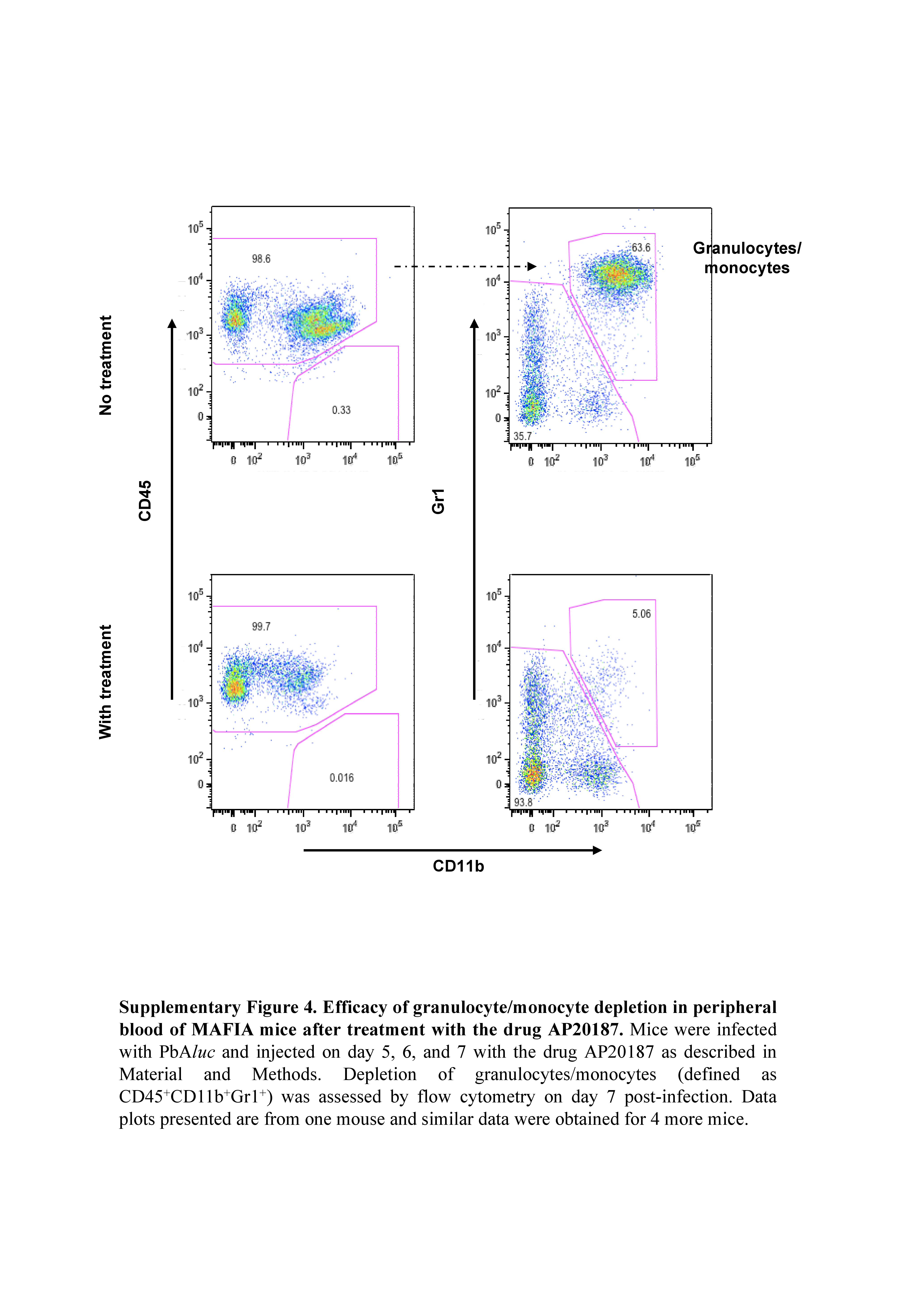

Supplement: Figure S4 — Efficacy of granulocyte/monocyte depletion in peripheral blood of MAFIA mice after treatment with the drug AP20187. Mice were infected with PbAluc and injected on day 5, 6, and 7 with the drug AP20187 as described in Material and Methods. Depletion of granulocytes/monocytes (defined as CD45+CD11b+Gr1+) was assessed by flow cytometry on day 7 post-infection. Data plots presented are from one mouse and similar data were obtained for 4 more mice. (TIFF) [file pone.0018720.s004.tif]

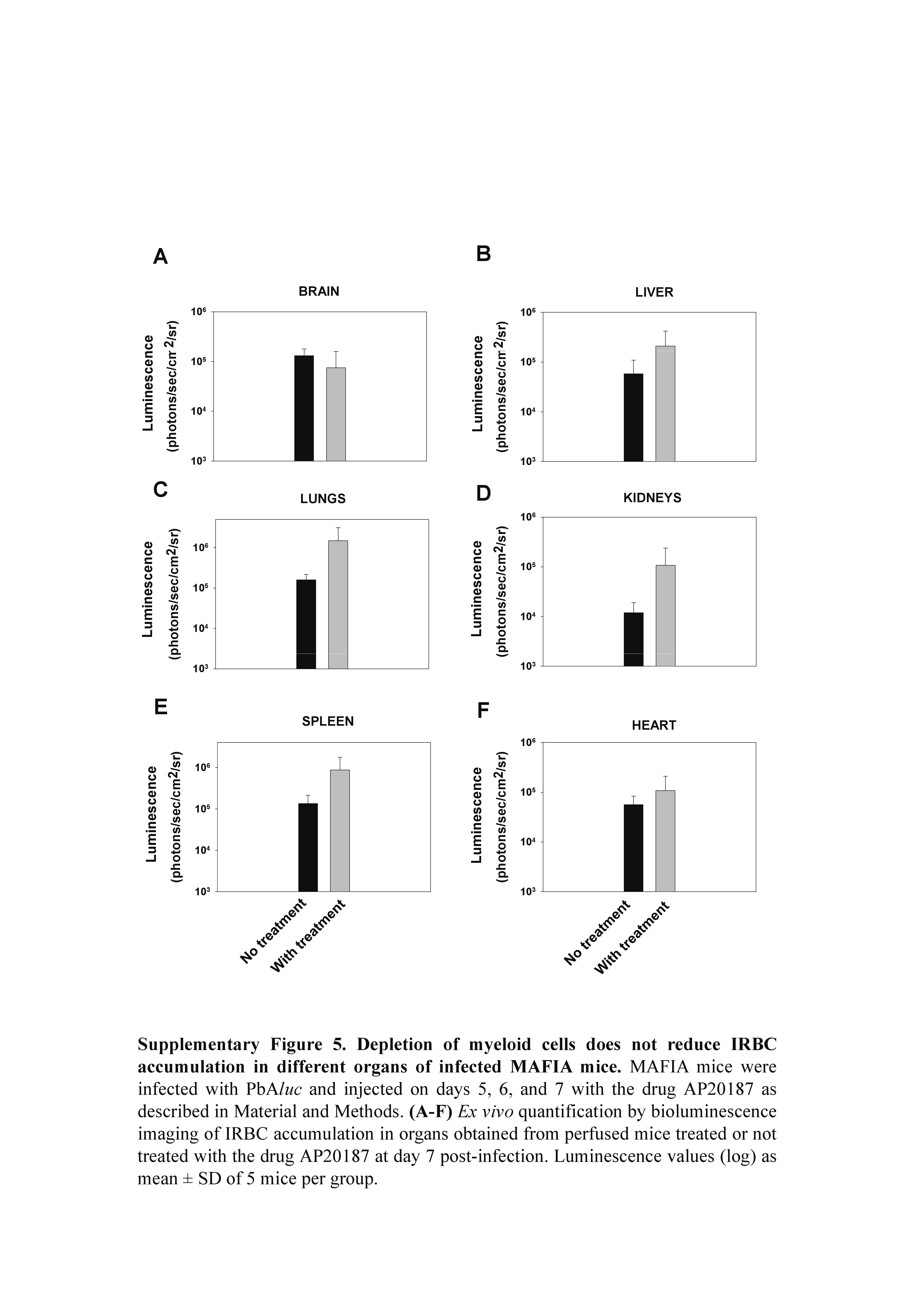

Supplement: Figure S5 — Depletion of myeloid cells does not reduce IRBC accumulation in different organs of infected MAFIA mice. MAFIA mice were infected with PbAluc and injected on days 5, 6, and 7 with the drug AP20187 as described in Material and Methods. (A–F) Ex vivo quantification by bioluminescence imaging of IRBC accumulation in organs obtained from perfused mice treated or not treated with the drug AP20187 at day 7 post-infection. Luminescence values (log) as mean ± SD of 5 mice per group. (TIFF) [file pone.0018720.s005.tif]

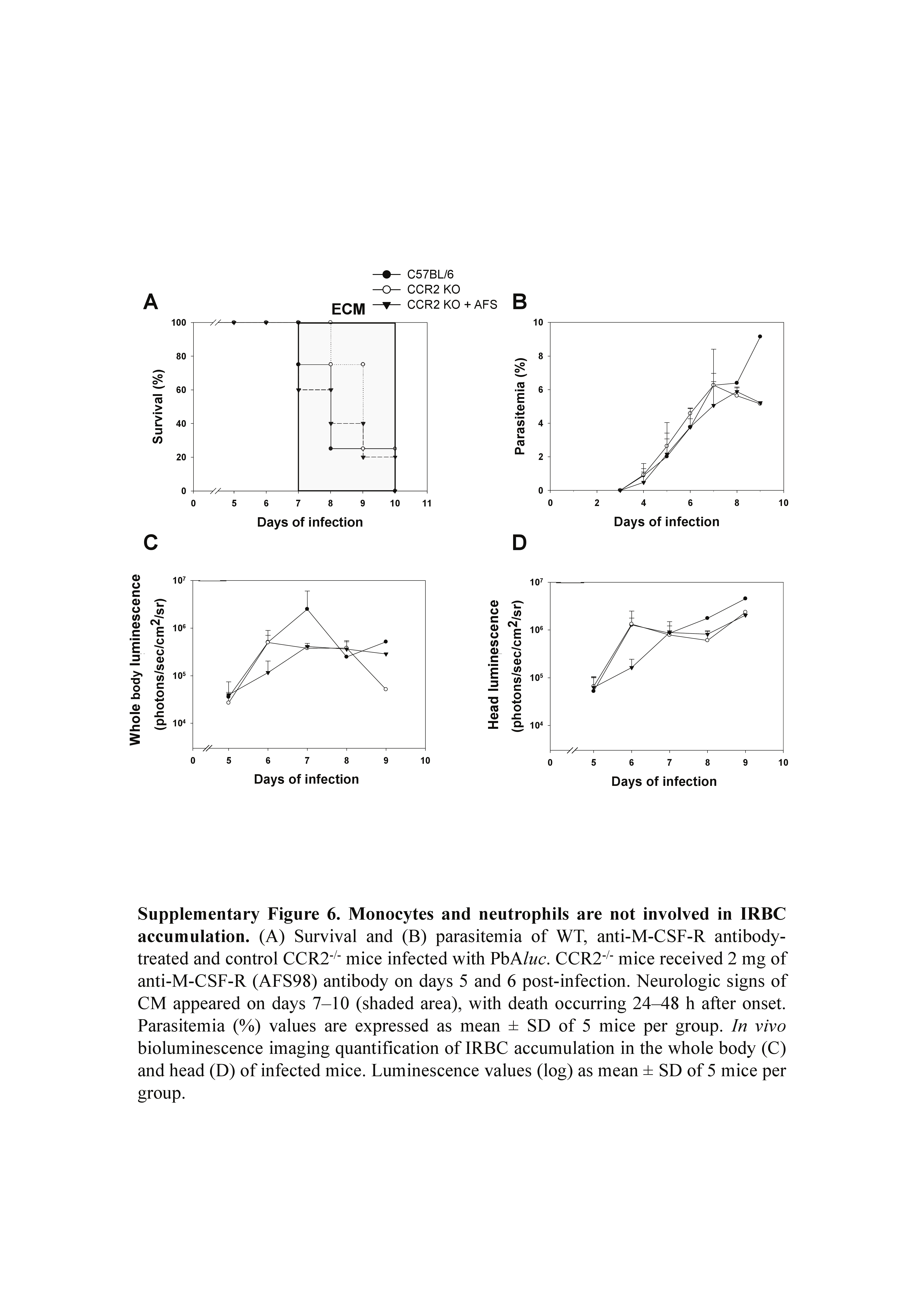

Supplement: Figure S6 — Monocytes and neutrophils are not involved in IRBC accumulation. (A) Survival and (B) parasitemia of WT, anti-M-CSF-R antibody-treated and control CCR2−/− mice infected with PbAluc. CCR2−/− mice received 2 mg of anti-M-CSF-R (AFS98) antibody on days 5 and 6 post-infection. Neurologic signs of CM appeared on days 7–10 (shaded area), with death occurring 24–48 h after onset. Parasitemia (%) values are expressed as mean ± SD of 5 mice per group. In vivo bioluminescence imaging quantification of IRBC accumulation in the whole body (C) and head (D) of infected mice. Luminescence values (log) as mean ± SD of 5 mice per group. (TIFF) [file pone.0018720.s006.tif]

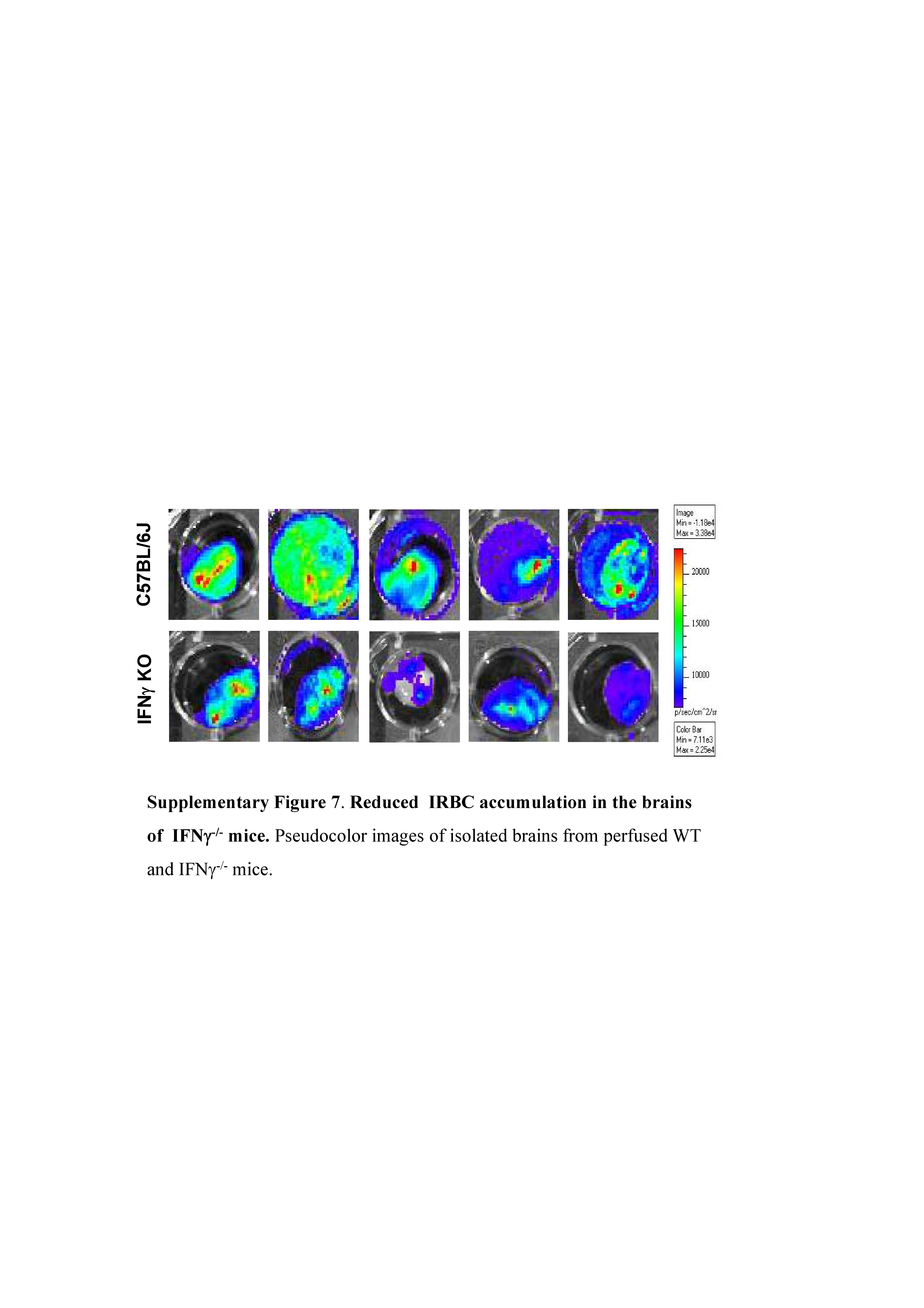

Supplement: Figure S7 — Reduced IRBC accumulation in the brains of IFN-γ−/− mice. Pseudocolor images of isolated brains from perfused WT and IFN-γ−/− mice at day 7 post-infection. (TIFF) [file pone.0018720.s007.tif]

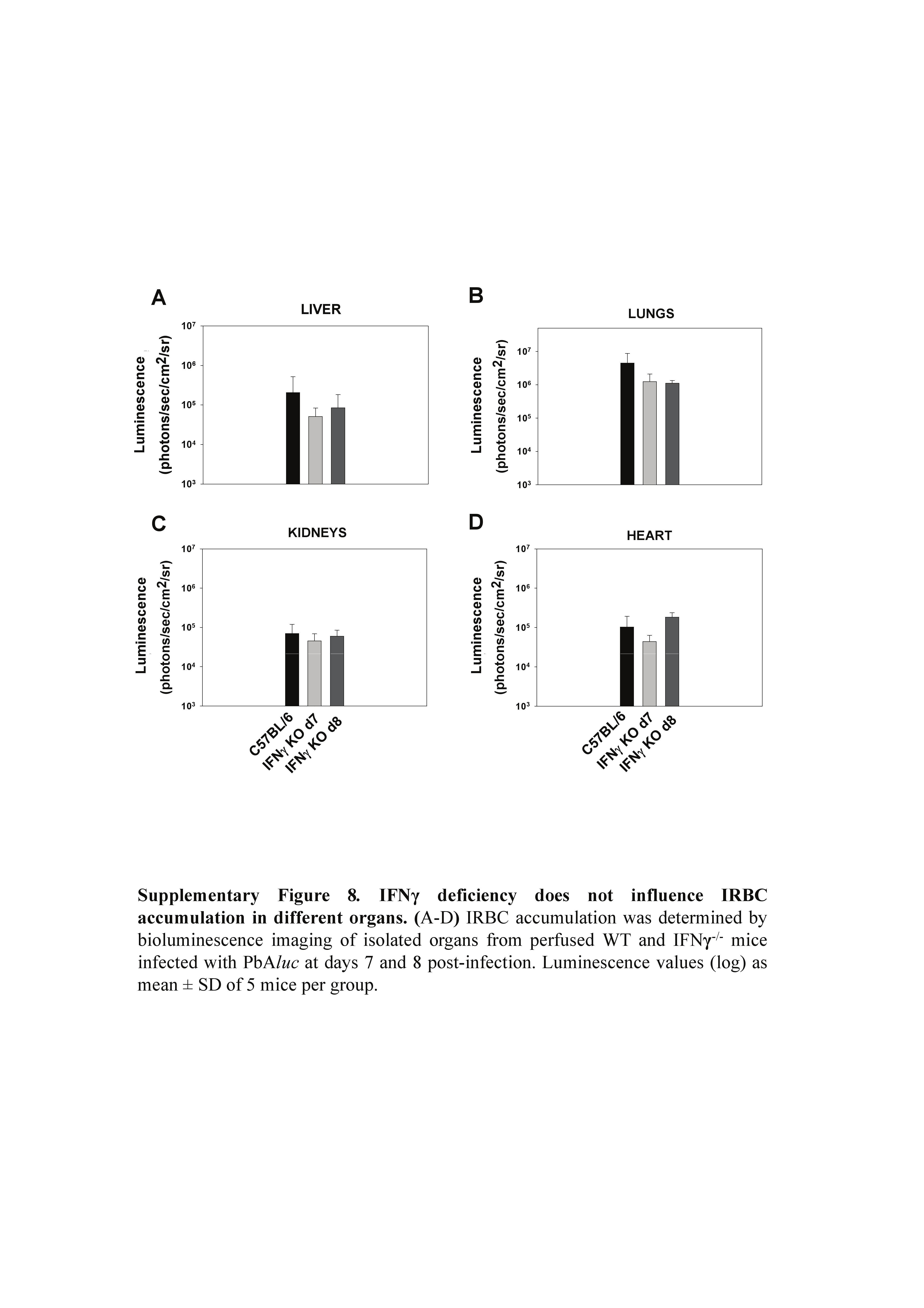

Supplement: Figure S8 — IFN-γ deficiency does not influence IRBC accumulation in different organs. (A–D) IRBC accumulation was determined by bioluminescence imaging of isolated organs from perfused WT and IFN-γ−/− mice infected with PbAluc at days 7 and 8 post-infection. Luminescence values (log) as mean ± SD of 5 mice per group. (TIFF) [file pone.0018720.s008.tif]

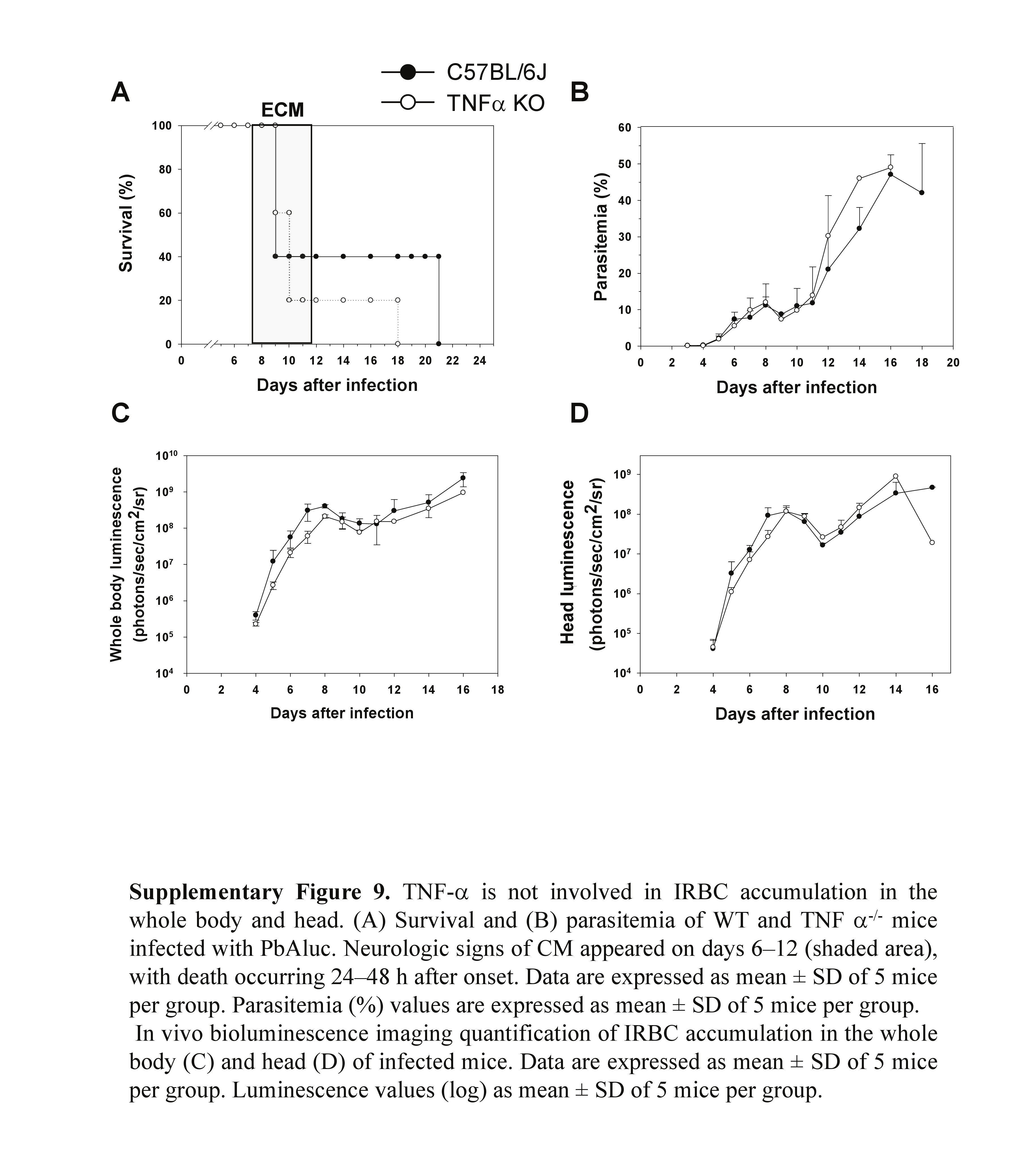

Supplement: Figure S9 — TNF-α is not involved in IRBC accumulation in the whole body and head. (A) Survival and (B) parasitemia of WT and TNFα−/− mice infected with PbAluc. Neurologic signs of CM appeared on days 6–12 (shaded area), with death occurring 24–48 h after onset. Data are expressed as mean ± SD of 5 mice per group. Parasitemia (%) values are expressed as mean ± SD of 5 mice per group. In vivo bioluminescence imaging quantification of IRBC accumulation in the whole body (C) and head (D) of infected mice. Data are expressed as mean ± SD of 5 mice per group. Luminescence values (log) as mean ± SD of 5 mice per group. (TIFF) [file pone.0018720.s009.tif]

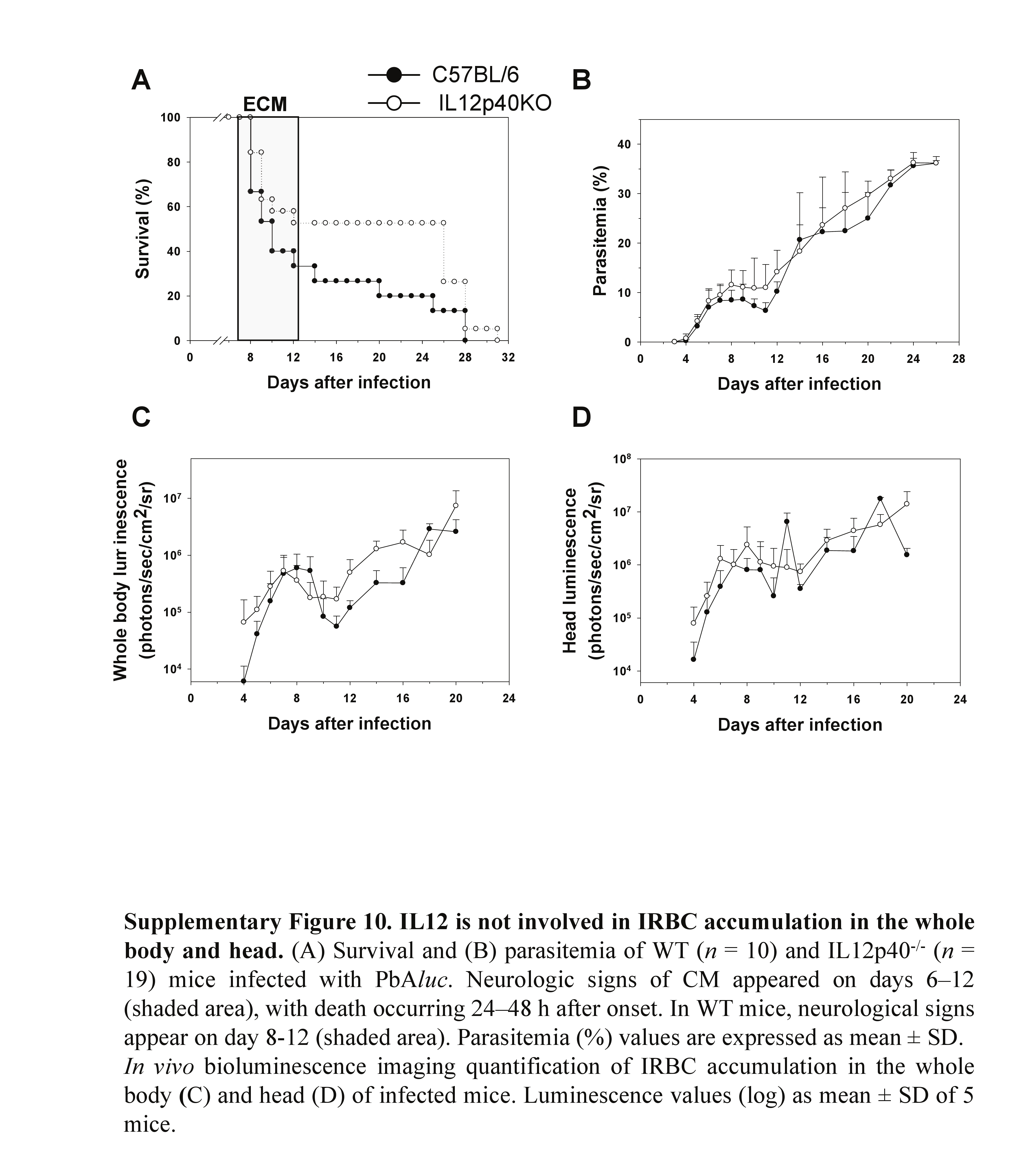

Supplement: Figure S10 — IL12 is not involved in IRBC accumulation in the whole body and head. (A) Survival and (B) parasitemia of WT (n = 10) and IL12p40−/− (n = 19) mice infected with PbAluc. Neurologic signs of CM appeared on days 6–12 (shaded area), with death occurring 24–48 h after onset. In WT mice, neurological signs appear on day 8–12 (shaded area). Parasitemia (%) values are expressed as mean ± SD. In vivo bioluminescence imaging quantification of IRBC accumulation in the whole body (C) and head (D) of infected mice. Luminescence values (log) as mean ± SD of 5 mice. (TIFF) [file pone.0018720.s010.tif]

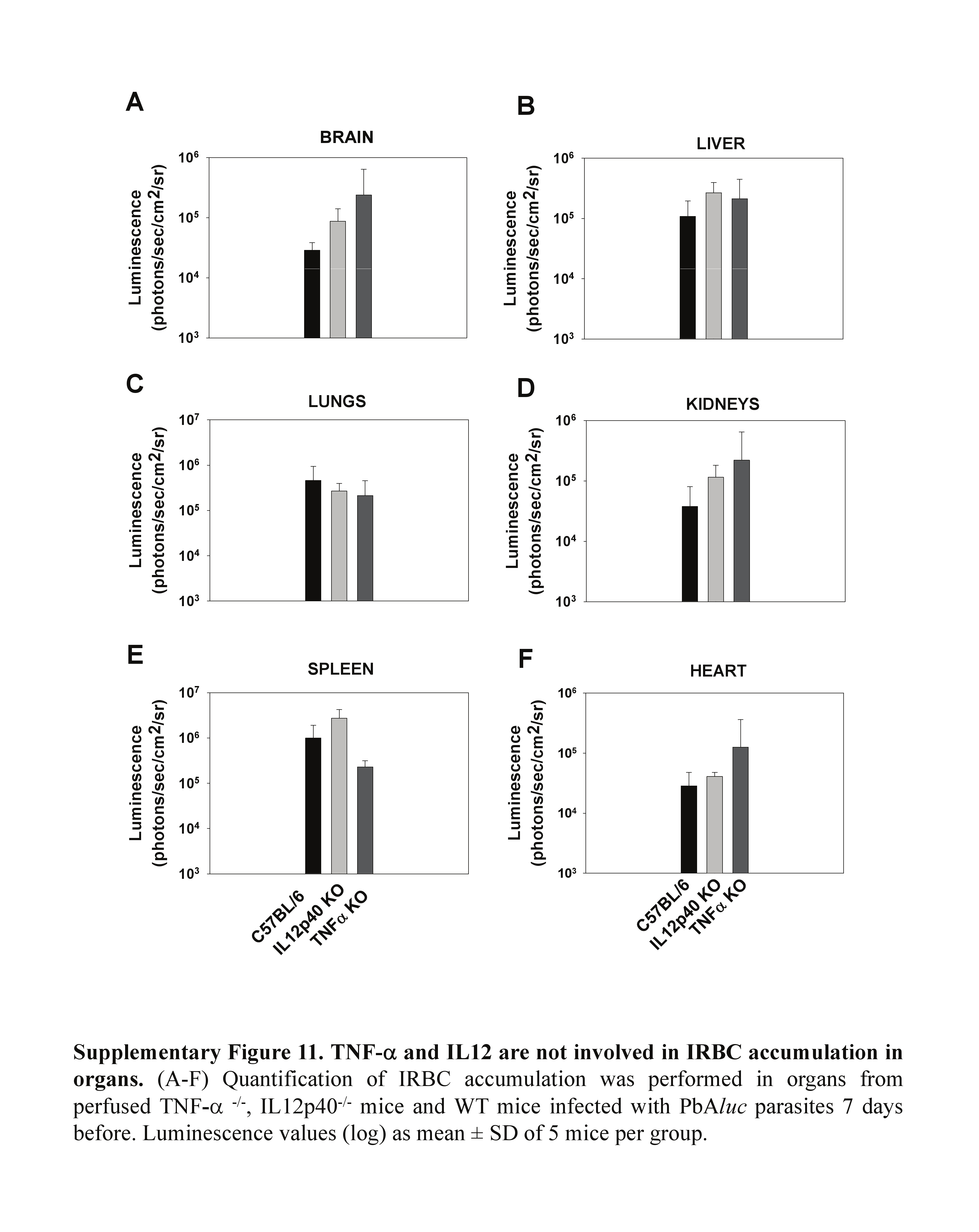

Supplement: Figure S11 — TNF-α and IL12 are not involved in IRBC accumulation in organs. (A–F) Quantification of IRBC accumulation was performed in organs from perfused TNF-α −/−, IL12p40−/− mice and WT mice infected with PbAluc parasites 7 days before. Luminescence values (log) as mean ± SD of 5 mice per group. (TIFF) [file pone.0018720.s011.tif]
